# Effects of Sucroferric Oxyhydroxide Compared to Lanthanum Carbonate and Sevelamer Carbonate on Phosphate Homeostasis and Vascular Calcifications in a Rat Model of Chronic Kidney Failure

**DOI:** 10.1155/2015/515606

**Published:** 2015-06-29

**Authors:** Olivier Phan, Marc Maillard, Hartmut H. Malluche, Jean-Christophe Stehle, Felix Funk, Michel Burnier

**Affiliations:** ^1^Department of Internal Medicine, Service of Nephrology and Hypertension, Centre Hospitalier Universitaire Vaudois (CHUV) and University of Lausanne, Lausanne, Switzerland; ^2^Division of Nephrology, Bone & Mineral Metabolism, University of Kentucky, Lexington, KY, USA; ^3^Division of Experimental Pathology, Institute of Pathology, Centre Hospitalier Universitaire Vaudois (CHUV), University of Lausanne, Lausanne, Switzerland; ^4^Vifor (International) Ltd., St. Gallen, Switzerland

## Abstract

Elevated serum phosphorus, calcium, and fibroblast growth factor 23 (FGF23) levels are associated with cardiovascular disease in chronic renal disease. This study evaluated the effects of sucroferric oxyhydroxide (PA21), a new iron-based phosphate binder, versus lanthanum carbonate (La) and sevelamer carbonate (Se), on serum FGF23, phosphorus, calcium, and intact parathyroid hormone (iPTH) concentrations, and the development of vascular calcification in adenine-induced chronic renal failure (CRF) rats. After induction of CRF, renal function was significantly impaired in all groups: uremic rats developed severe hyperphosphatemia, and serum iPTH increased significantly. All uremic rats (except controls) then received phosphate binders for 4 weeks. Hyperphosphatemia and increased serum iPTH were controlled to a similar extent in all phosphate binder-treatment groups. Only sucroferric oxyhydroxide was associated with significantly decreased FGF23. Vascular calcifications of the thoracic aorta were decreased by all three phosphate binders. Calcifications were better prevented at the superior part of the thoracic and abdominal aorta in the PA21 treated rats. In adenine-induced CRF rats, sucroferric oxyhydroxide was as effective as La and Se in controlling hyperphosphatemia, secondary hyperparathyroidism, and vascular calcifications. The role of FGF23 in calcification remains to be confirmed.

## 1. Introduction

Cardiovascular disease (CVD) is a major cause of mortality in patients with chronic kidney disease (CKD) [1]. Several factors were identified in this process; elevated serum phosphorus and FGF23 levels in particular are recognized to be associated with CVD in patients with CKD [2–5]. Current therapy focuses on decreasing serum phosphorus levels in CKD using phosphate binders and nutritional consulting. Sucroferric oxyhydroxide (PA21) is a new iron (III)-oxyhydroxide containing compound in which addition of carbohydrates prevents iron oxyhydroxide from aging and maintains its phosphate-binding capacity [6, 7].

FGF23 is a phosphaturic hormone that has an important role in phosphate homeostasis [8]. FGF23 is produced by osteocytes and directly affects the kidney by downregulating the production of 1,25-vitamin D3 and the expression of 2a and 2c sodium-phosphate cotransporters in response to phosphate overload [9]. FGF23 secretion from bone increases as renal function declines. Phosphate retention occurs early in the course of renal failure. FGF23 increases urinary phosphate excretion and can be viewed as an adaptive response to an elevated phosphate load. High FGF-23 levels are associated with death and cardiovascular events in subjects with CKD [10]. To date, there is little information on the effects of phosphorus lowering by pharmacologic means on plasma FGF23 levels. Abnormalities in mineral metabolism also appear early in the course of CKD and result in clinically relevant consequences such as vascular calcification. It has recently been established that levels of FGF23 are increased in early CKD [11].

We have previously shown that sucroferric oxyhydroxide (PA21) is as effective as calcium carbonate, the traditional phosphate binder, for controlling serum phosphorus levels, and is superior in preventing the development of vascular calcifications in uremic rats [12]. The aim of this study was to compare the effects of sucroferric oxyhydroxide (PA21) with two other noncalcium phosphate binders, lanthanum carbonate and sevelamer carbonate, on concentrations of serum FGF23, phosphorus, calcium, and intact parathyroid hormone (iPTH), and in particular to investigate its potential effect on the development of vascular calcifications in an adenine-induced CKD model in rats.

## 2. Methods

### 2.1. Experimental Design

The procedures for the care and use of experimental animals and the protocols were submitted to and accepted by the local veterinary authorities, University of Lausanne. Male Wistar rats (Charles River Laboratories, L'Arbresle, France) were housed (3 rats/cage) in polycarbonate cages in a pathogen-free, temperature-controlled (25°C) facility with a strict 12-hour light/dark cycle. The rats had free access to chow diet and water [12].

As previously described, to induce chronic renal failure (CRF), 10-week-old rats were fed a diet containing 0.75% adenine and a high phosphorus content (1.3% phosphorus, 1.06% calcium, 1000 IU/kg vitamin D3, and 23% protein) (Kliba Nafag 3200, Provimi Kliba AG, Kaiseraugst, Switzerland, supplemented with phosphorus and calcium) for 4 weeks [12]. After this time, adenine was withdrawn from the high-phosphorus diet and blood sampling was performed at the tail vein [12].

To compare the effects of the phosphate binders, the same concentration (1% in the diet) of metal or resin, respectively, was applied for each phosphate binder: 5% sucroferric oxyhydroxide (PA21) was used to match with 1% iron, 2% lanthanum carbonate to match with 1% lanthanum, and 1.5% Renvela to match with 1% of sevelamer carbonate. The mixing of the binder with the diet was a two-step process: firstly, a dry mixture was prepared and stored protected from air and light; secondly, it was hydrated with the same volume of water and homogenized before offering the diet to the animals [12].

The uremic rats were randomly assigned to one of the following four CRF groups: (1) a control group, untreated throughout the experiments (*n* = 22); (2) a group treated with sucroferric oxyhydroxide (PA21) at 5% (CRF PA21; *n* = 20); (3) a group treated with lanthanum carbonate at 2% (CRF La; *n* = 20); and (4) a group treated with sevelamer carbonate at 1.5% (CRF Se; *n* = 20).

At sacrifice, after 4 weeks of treatment, blood was sampled by cardiac puncture for determination of serum calcium, phosphorus, creatinine, alkaline phosphatase, iPTH, hematocrit, and ferritin [12]. In addition, urine spot collection was performed at sacrifice for all rats. After blood sampling, animals were euthanized and perfused with formalin. Thereafter, aorta, carotid, and femoral arteries were harvested.

### 2.2. Phosphate Binders

Sucroferric oxyhydroxide (PA21), lot 097101A11, was provided by Vifor (International) Inc., St. Gallen, Switzerland. Lanthanum(III) carbonate hydrate, lot C16W033, was purchased from Alfa Aesar GmbH & Co, Karlsruhe, Germany, and sevelamer carbonate (Renvela), lot D0020B01, was from Genzyme, Naarden, The Netherlands.

### 2.3. Biochemical Analyses

Serum and urine creatinine, calcium and phosphorus, and alkaline phosphatase concentrations were measured using standard colorimetric method (Cobas Mira, Roche, Basel, Switzerland). Serum iPTH and ferritin were measured using commercial ELISA kits specific for the full-length bioactive rat 1–84 iPTH and for rat ferritin (both kits from Alpco Diagnostics, Salem, NH, USA). FGF23 was assessed using a commercial full-length ELISA kit specific for rat FGF23 (Millipore; Billerica, MA, USA). Hematocrit was measured in triplicate immediately at the time of sacrifice, using microfuge centrifugation (3 min at 12,000 rpm) of a small blood droplet within a glass capillary.

### 2.4. Calcification Measurements

The carotid arteries, as well as abdominal and thoracic aorta, were removed, fixed in neutral buffered formalin, and cut into rings embedded upright in the same paraffin block. Sections (4 *μ*m thickness) were stained using von Kossa's method [12–15]. Vascular calcifications were evaluated histomorphometrically at a magnification of 40x and were assessed in a blinded manner on random sections of aorta. Morphologic image-processing algorithms were used for computer-assisted automated quantitative measurement of calcification from vessel sections (Leika image-analysis software), revealed by the von Kossa's silver nitrate staining. Data were expressed as the relative proportion (%) of calcified area (*C*) of the aorta media to total surface area (*T*) of each ring (*C*/*T*). To avoid any misinterpretation, the aorta rings were evaluated only if the entire surface of the ring was present.

### 2.5. Statistical Analysis

Results are expressed as mean ± SEM. Data derived from the different experimental groups were compared using one-way analysis of variance and the Bonferroni post hoc test, with the exception of the FGF23 data (which do not follow a Gaussian distribution), for which the median and range are reported; a nonparametric analysis (Kruskal-Wallis test followed by Mann-Whitney post hoc test) was used for this dataset. Chi-square analysis was used to compare the data on vascular calcifications. A *p* value of <0.05 was considered significant, and all computations were done using SPSS version 7.0 (SPSS, Inc, Chicago, IL, USA).

## 3. Results

### 3.1. Mortality

Of the total 82 CRF rats, seven had to be euthanized 1 week after cessation of adenine feeding due to malnutrition (weight < 160 g) and severe uremia (4/22 in the control group, 1/20 in the sucroferric oxyhydroxide group, and 2/20 in the lanthanum carbonate group).

### 3.2. Biochemical Analyses

After 4 weeks of adenine administration to induce renal failure, and before starting the phosphate-binder treatment, mean body weight and concentrations of serum creatinine and calcium and phosphate concentrations were similar in all CRF groups (Table 1).

**Figure 1:**
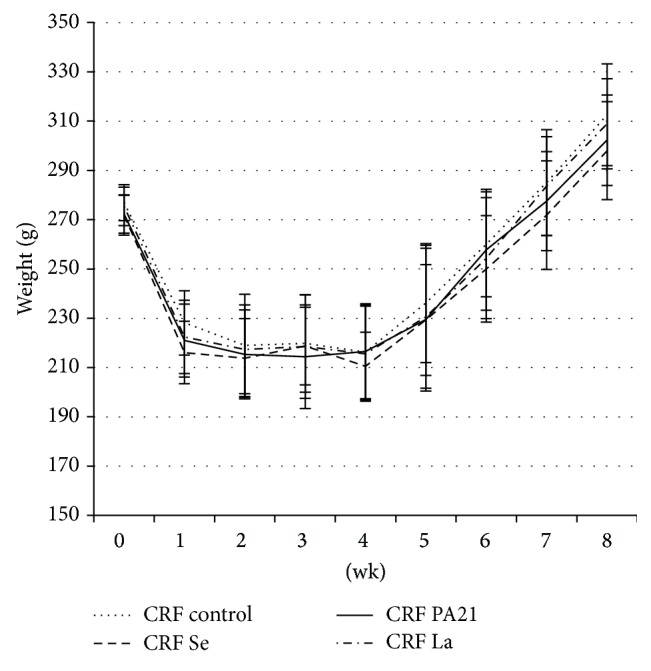
Change in body weight during the study.

There was no difference in body weight of CRF rats between time of randomization and end of the study (Figure 1).

Each animal was weighed weekly during the study. Adenine was started at Week 0 for 4 weeks; phosphate binders were started at Week 4 for 4 weeks after withdrawal of adenine. Rats were sacrificed on the first day of Week 8. There was no statistical difference between the groups.

After 4 weeks of phosphate-binder treatment, serum creatinine concentrations and also body weights were similar in all CRF groups (Table 2).

After 4 weeks of treatment with the three phosphate binders, a significant reduction in serum phosphorus was observed (Table 2). Serum calcium concentration was higher in the uremic rats treated by sevelamer and lanthanum carbonate. CRF control rats also developed marked hyperparathyroidism, as shown by elevated iPTH levels at time of sacrifice (Table 2). iPTH was reduced to a similar extent by the three phosphate binders (*p* < 0.05), while serum alkaline phosphatase levels were only slightly reduced in these groups although the difference was not statistically significant (Table 2). Phosphaturia was dramatically decreased in all three groups (*p* < 0.05) (Table 3). Hematocrit levels were significantly lower in the sevelamer carbonate and lanthanum carbonate groups compared with the CRF control group (Table 2).

**Figure 2:**
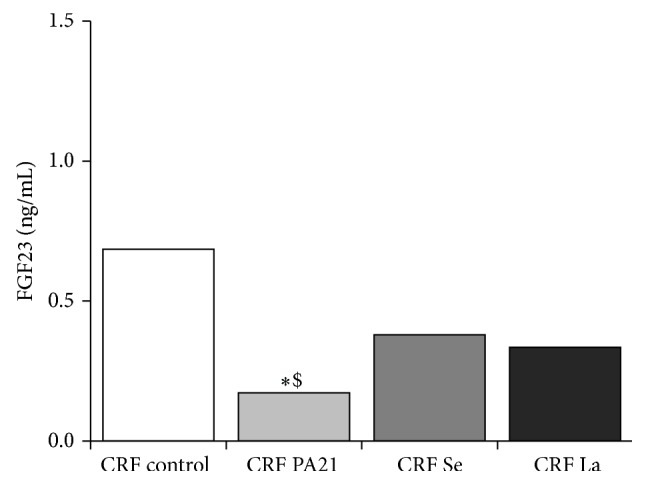
Median serum FGF23 concentration after 4 weeks of binder treatment.

Uremia was associated with increased serum FGF23 concentration in the CRF control group. Serum FGF23 concentration were lower in all three groups treated with phosphate-binders, but this was statistically significant only in the case of sucroferric oxyhydroxide (PA21) (Figure 2).

The effect of sucroferric oxyhydroxide (PA21) and other phosphate binder on FGF23 concentrations was evaluated after 4 weeks of treatment. Serum FGF23 was decreased (*p* < 0.05) in animals in the CRF PA21 group (*n* = 19, median 0.2 ng/mL, range 0.1–7.6), compared with the CRF control group animals (*n* = 18, median, 0.7 ng/mL, range 0.1–9.7). Serum FGF23 in the CRF PA21 group was also lower (*p* < 0.05) than in the CRF Se group (*n* = 19, median 0.4, range 0.1–10.6). In contrast, there were no differences in serum FGF23 between the CRF control, Se, and La groups (La: *n* = 19, median 0.3 ng/mL, range 0.1–9.1).

### 3.3. Vascular Calcifications

**Figure 3:**
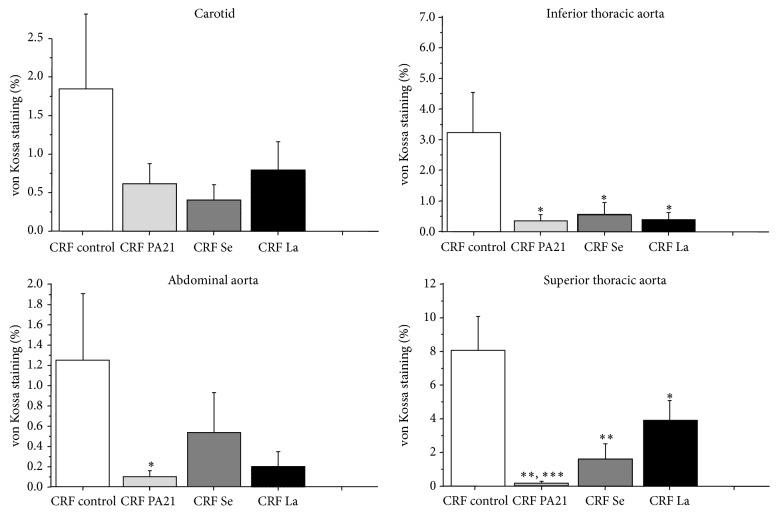
Vascular calcifications in carotid artery, abdominal aorta, and inferior and superior part of thoracic aorta in uremic controls and phosphate-binder groups. ^*∗*^ *p* < 0.05 versus CRF control, ^*∗∗*^ *p* < 0.001 versus CRF control, and ^*∗∗∗*^ *p* < 0.05 versus CRF La.

**Figure 4:**
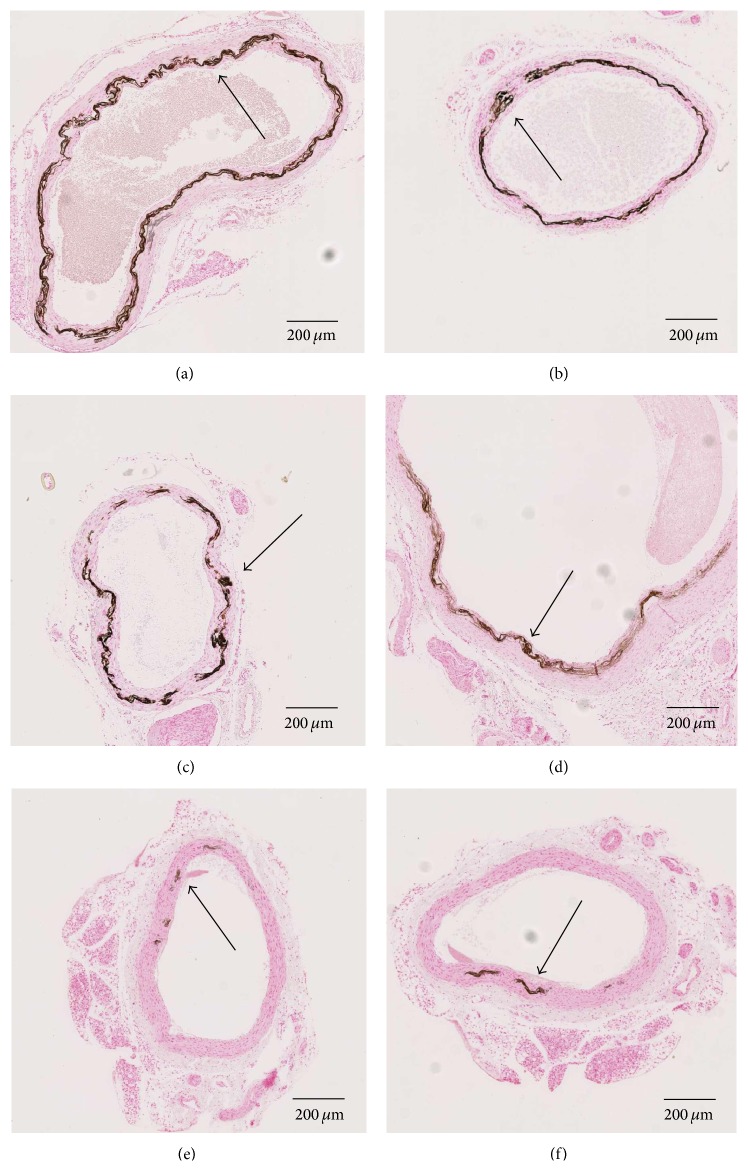
von Kossa stains of the vascular calcifications of adenine fed rats after 4 weeks of treatment. Vascular calcifications were evaluated by von Kossa staining and histomorphometrically with a magnification of 40. (a) Superior thoracic aorta, (b) carotid, and (c) abdominal aorta from CRF control rats. (d) Superior thoracic aorta, (e) carotid, and (f) abdominal aorta from CRF rats treated by sucroferric oxyhydroxide (PA21).

Vascular calcifications were observed in more than 70% of the uremic control rats. Vascular calcifications were evaluated at different sites: (a) carotids, (b) inferior and superior part of the thoracic aorta, and (c) abdominal aorta. Vascular calcifications of the thoracic aorta (inferior and superior part) were significantly decreased in all three phosphate binder groups (Figures 3 and 4 and Table 4). Vascular calcifications of the abdominal aorta were only decreased in the sucroferric oxyhydroxide (PA21) group and PA21 was more effective than lanthanum carbonate in preventing calcifications in the superior part of the thoracic aorta (Figures 3 and 4 and Table 4).

## 4. Discussion

This is the first study to compare sucroferric oxyhydroxide with sevelamer carbonate and lanthanum carbonate in an animal model. It demonstrates that sucroferric oxyhydroxide is as effective as sevelamer carbonate and lanthanum carbonate in decreasing serum phosphorus and iPTH concentrations and reducing the progression of vascular calcifications.

The weight of the animals in all CRF groups decreased during the 4 weeks of adenine administration and increased after adenine was withdrawn. A body weight decrease in adenine-induced CRF animals has been described in previous studies [12, 16] and is probably due to development of uremia and absence of appetite during the administration of adenine.

Based on the results of serum and urinary phosphorus levels, sucroferric oxyhydroxide was shown to be equally as effective in controlling hyperphosphatemia as sevelamer carbonate and lanthanum carbonate. Secondary hyperparathyroidism was similarly reduced by all three binders. Vascular calcifications of thoracic aorta were significantly decreased by the three phosphate binders to a similar extent by the control of hyperphosphatemia and hyperparathyroidism [12, 17, 18]. Less calcification in the upper part of the thoracic aorta was observed in sucroferric oxyhydroxide-treated animals than in those treated with lanthanum carbonate. Several studies have reported that the risk of calcification increases in the presence of hypercalcemia [19, 20]. The absence of significant serum hypercalcemia concentration in the sucroferric oxyhydroxide-treated animals could provide a potential explanation for the reduced vascular calcification. London et al. have suggested that the hypercalcemia associated with higher calcium phosphate product levels may worsen cardiovascular events in the uremic population, through a progressive increase in calcium deposition in the coronary arteries and heart valves [19]. More recently, in a study including 384 dialysis patients, Noordzij et al. reported that hypercalcemia and hyperparathyroidism were associated with an increased risk of progression of vascular calcifications [20]. Additional influences, such as hemodynamic factors relating to specific anatomic sites of aorta, could lead to a differential progression of atherosclerosis and intima-media calcification [21–23]. In this study we did not include measurement of hemodynamic parameters and thus cannot address a potential contribution of differences in hemodynamics. Interestingly, the vascular calcifications of the aorta differed, in regard to their anatomical position. Some studies demonstrate a high level of vascular calcification in this adenine uremic rat model [24–26]; vascular calcifications were presents in 70% of uremic control rats in our study. Conversely, Neven et al. have reported that only 50% of uremic rats developed vascular calcifications [16]. These differences could be explained by relative differences between the studies concerning the concentration and the duration of the adenine, the adjunction of vitamin D, and the calcium and phosphorus concentration of the diet which influence the degree of calcifications and point the difficulties to obtain the adequate uremic animal model of vascular calcifications. More recently, a low protein diet or the administration of warfarin seems to increase the rate of vascular calcifications. Warfarin inhibits matrix Gla protein, a vitamin K-dependent protein that counteracts artery calcification [27].

High FGF23 levels are associated with death and cardiovascular events in CKD patients [28] and may be explained by the direct effect of FGF23 on the heart, which was reported by Faul et al. [3]. In the early stages of CKD, circulating FGF23 levels increase with declining renal function as a physiological compensation to stabilize serum phosphorus levels [29, 30]. Results from some clinical studies suggest there is an association between elevated FGF23 and vascular calcification [31]. Recently, it was also shown that FGF23 enhanced phosphate-induced calcification in Klotho-overexpressing vascular smooth muscle cells and increased osteoblastic marker expression [32]. However, conflicting results have been reported in the literature. The relationship between FGF23 and vascular calcification remains a matter of debate [33]. In one study, high serum levels of FGF23 were reported to be associated with more severe vascular calcification in CKD patients [34], while another study involving non-CKD patients observed a negative association between FGF-23 and the presence of atherosclerotic lesions [35]. Most (though not all) recent studies have demonstrated an involvement of both Klotho and FGF23 in vascular calcifications, via their interaction or through separate individual effects [33, 36]. Their precise roles in vascular calcification will require further study.

Our results confirmed results of* in vitro* and preclinical investigations that sucroferric oxyhydroxide is an effective phosphate binder. Serum phosphorus and iPTH decreased rapidly with the three noncalcium phosphate binders. But, this is the first study witch evaluated the effect of sucroferric oxyhydroxide on vascular calcifications compared with lanthanum carbonate or sevelamer carbonate. The three binders had a positive influence of the progression of the vascular calcification. Interestingly, a slightly greater effect on the superior part of thoracic aorta was observed with sucroferric oxyhydroxide, compared with the other two phosphate binders. One could speculate that, after a longer period of treatment, lanthanum carbonate or sevelamer carbonate might also similarly affect the level of FGF23. In a previous study, we demonstrated that, in comparison with sucroferric oxyhydroxide, the traditional calcium carbonate phosphate binder decreased iPTH and phosphorus concentration but not FGF23 levels. We also showed that, at the same time, vascular calcifications were less well prevented by calcium carbonate, in comparison with sucroferric oxyhydroxide [12]. For other noncalcium phosphate binders, the results reported in the literature are conflicting, with respect to variation of serum FGF23 levels. Maizel et al. reported that sevelamer hydrochloride improved aortic systolic expansion rate, pulse-wave velocity, and diastolic function, but serum FGF23 levels were reduced after 14 weeks of treatment [37]. In contrast, in a different animal model, Nagano et al. reported that sevelamer effectively inhibited the increase in serum FGF23 and PTH while calcification was not evaluated [38]. In contrast to previous studies in normophosphatemic subjects, one study attempted to investigate whether sevelamer carbonate could lower FGF23 levels in patients with CKD (Stages 3–5) and hyperphosphatemia [39]. Although serum phosphorus reduction was achieved, no significant reduction in plasma FGF23 levels was seen. The novel aspect of the study was that FGF23 was reduced as early as three months and did not require prolonged therapy. Our study provides the first data on sucroferric oxyhydroxide and FGF23 in rats. Further studies are needed to show whether an early decrease of FGF23 by sucroferric oxyhydroxide can predict a slower progression of vascular calcification in CKD.

## 5. Conclusions

Administration of sucroferric oxyhydroxide, a new iron-based noncalcium phosphate binder, to rats with adenine-induced CRF was found to be as effective in controlling hyperphosphatemia, secondary hyperparathyroidism, and vascular calcification as sevelamer and lanthanum carbonate.

## Figures and Tables

**Table 1:** Serum biochemistry, prior to binder treatment.

CRF group/binder type	*N*	Weight g	Creatinine *µ*mol/L	Ca mmol/L	P mmol/L
CRF control	18	216 ± 4	251 ± 17.7	2.27 ± 0.40	4.83 ± 0.28
CRF PA21	19	217 ± 4	239 ± 19.0	2.44 ± 0.04	4.75 ± 0.32
CRF Se	20	211 ± 3	229 ± 16.2	2.42 ± 0.06	4.44 ± 0.23
CRF La	18	216 ± 4	233 ± 10.6	2.41 ± 0.03	4.62 ± 0.21

Ca, calcium; La, lanthanum carbonate; P, phosphorus; Se, sevelamer carbonate; PA21, sucroferric oxyhydroxide. Values shown are mean ± SE.

**Table 2:** Serum biochemistry, after 4 weeks of binder treatment, at time of sacrifice.

	*N*	Weight g	Creatinine *μ*mol/L	Ca mmol/L	P mmol/L	iPTH pg/mL	AP mmol/L	Ht %	Ferritin ng/mL	FGF23 ng/mL
CRF control	18	313 ± 5	144 ± 11	2.32 ± 0.05	3.30 ± 0.29	3567 ± 593	430 ± 26	26.7 ± 0.5	2359 ± 281	0.7 (0.1–9.7)
CRF PA21	19	302 ± 4	141 ± 10	2.45 ± 0.03	2.06 ± 0.06^a^	1459 ± 242^a^	363 ± 19	26.4 ± 0.5	2185 ± 173	0.2 (0.1–7.6)^a^
CRF Se	20	298 ± 4	147 ± 11	2.48 ± 0.03^a^	2.51 ± 0.12^a^	1569 ± 238^a^	367 ± 19	21.9 ± 0.5^a^	2188 ± 134	0.4 (0.1–10.6)
CRF La	18	309 ± 4	140 ± 7.9	2.52 ± 0.03^a^	2.24 ± 0.07^a^	1360 ± 170^a^	379 ± 29	22.7 ± 0.5^a^	2361 ± 184	0.3 (0.1–9.1)

Ca, calcium; Ht, hematocrit; iPTH, intact parathyroid hormone; La, lanthanum carbonate; AP, alkaline phosphatase; P, phosphorus; Se, sevelamer carbonate; PA21, sucroferric oxyhydroxide. ^a^ *p* < 0.05 versus CRF control. Values shown are mean ± SE except for FGF23 (median and range).

**Table 3:** Urinary values at sacrifice.

CRF group/binder type	*N*	Ca/creat. U mmol/mmol	P/creat. U mmol/mmol
CRF control	18	1.45 ± 0.17	17.8 ± 1.91
CRF PA21	16	1.12 ± 0.12	4.31 ± 0.61^a^
CRF Se	20	1.44 ± 0.12	10.1 ± 0.73^a^
CRF La	17	1.45 ± 0.12	7.88 ± 0.61^a^

Ca, calcium; La, lanthanum carbonate; P, phosphorus; Se, sevelamer carbonate; PA21, sucroferric oxyhydroxide. ^a^ *p* < 0.05 versus CRF control. Values shown are mean ± SE.

**Table 4:** Impact of phosphate binder treatment on vascular calcifications of carotid artery, abdominal aorta, and inferior and superior part of thoracic aorta.

	Carotid	Abdominal aorta	Inferior thoracic aorta	Superior thoracic aorta
CRF PA21	No	Yes	Yes	Yes
CRF Se	No	No	Yes	Yes
CRF La	No	No	Yes	Yes

## Abbreviations

CKD: Chronic kidney disease
CRF: Chronic renal failure
FGF23: Fibroblast growth factor 23
iPTH: Intact parathyroid hormone
La: Lanthanum carbonate
Se: Sevelamer carbonate
PA21: Sucroferric oxyhydroxide.
